# A case report of cryptococcal meningoencephalitis presenting as uveitis

**DOI:** 10.1007/s12348-012-0076-8

**Published:** 2012-05-07

**Authors:** Nikita Abhyankar, Showrob Patwary, Swathi Panneerselvam, Nirodhini Narendran, Yit C. Yang

**Affiliations:** Wolverhampton Eye Infirmary, New Cross Hospital, Wolverhampton, West Midlands WV10 0QP UK

## Introduction

In any patient with a first attack of posterior uveitis, it is important to differentiate whether the underlying cause is infective or non-infective in nature. However, when patients with a history of non-infective posterior inflammation present with recurrent symptoms of a flare-up, there is naturally a much lower suspicion of an infective aetiology and immunosuppression is usually commenced for the ‘flare-up’. We present a case of infective posterior uveitis due to a rare pathogen occurring in a patient with a known history of sarcoid uveitis which was initially misdiagnosed and treated as a ‘flare-up’.

## Case report

A 60-year-old male farmer with previous history of bilateral anterior and posterior uveitis, secondary to sarcoidosis, presented with new symptoms of visual blurring in the left eye, headaches and dizziness. Prior to this episode, he had been under regular follow-up for flare-ups of posterior uveitis affecting one or both eyes over a 10-year period. His episodes of inflammatory flare-ups have typically responded well to short courses of systemic corticosteroid therapy. The most recent flare-up, prior to this current episode 4 months earlier, was successfully treated with oral prednisolone with recovery to visual acuities of 6/6 right and 6/6 left.

**Fig. 1:**
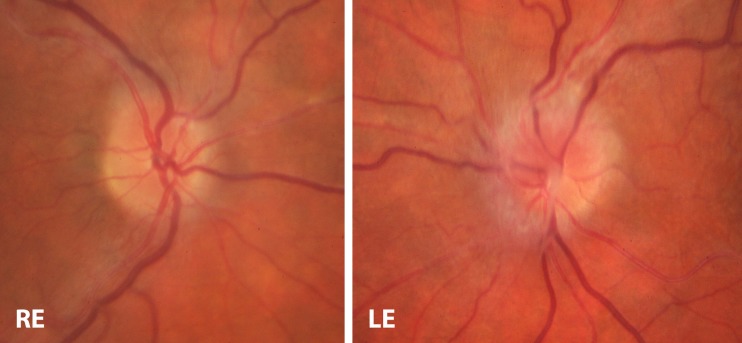
Colour fundus photograph of both eyes. Left eye shows optic disc swelling and epiretinal membrane. Right eye shows healthy optic nerve

On this presentation, his visual acuities were 6/6 right and 6/9 left. Colour vision was normal in the right eye (15/17), but was reduced in the left eye to 1/17 (Ishihara test). Both anterior segments were clear, without any signs of anterior uveitis. Posterior segment examination showed no signs of vitritis in either eye, but in the left eye there was a preexisting epiretinal membrane and new optic disc swelling (Fig. 1). Systemic examination was normal and he was apyrexial.

At this stage, several possibilities were being considered, including inflammatory flare-up of ocular sarcodosis with optic nerve involvement, neurosarcoid, or infiltrative or compressive optic neuropathy. CT and MRI scans of the head and brain showed no radiological signs of inflammatory or space-occupying lesions in the left optic nerve and chiasma. As there was no evidence to suggest an infective cause at that stage, he was commenced on 60 mg/day of oral prednisolone.

Initially, his left eye improved slightly to 6/6, but reduced colour vision persisted and headaches worsened, accompanied by transient confusion and an episode of slurred speech. The prednisolone dosage was briskly tapered and he was admitted into a hospital for further investigations. Because of the poor response to corticosteroid therapy and the appearance of new neurological symptoms, other neurological conditions were being considered, including tuberculous meningitis, fungal meningitis, prion disease and neurosarcoidosis. He was still apyrexial and initial blood investigations and infection screen (including VDRL, HIV and AFB) were all normal.

**Fig. 2:**
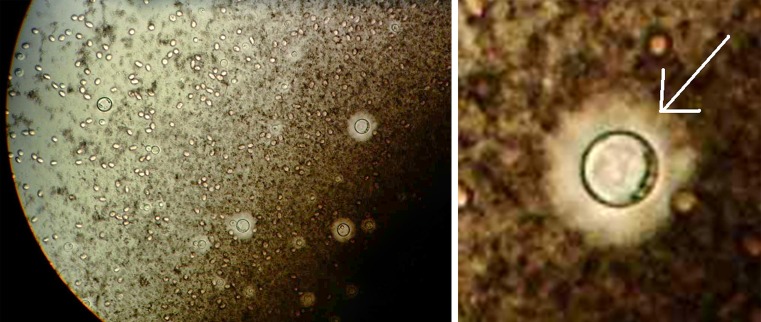
India ink stain for cryptococcal antigen

After several unsuccessful attempts at lumbar puncture, a CT-guided lumbar puncture yielded a sufficient sample which, on culture, grew a cryptococcal-like species which stains positive with India ink (silhouette indicating the presence of the polysaccharide capsule of *Cryptococcus* species; Fig. 2). The organism in this sample was confirmed as *Cryptococcus neoformans* using the latex agglutination test, which is superior to the India ink technique and is specific for the polysaccharide antigens of the encapsulated *neoformans* species [1].

The patient was thus diagnosed with meningoencephalitis secondary to *C. neoformans* infection and commenced on two weeks of intravenous antifungal therapy of amphotericin 4 mg/kg once a day and flucytosine 25 mg/kg four times a day, followed later by oral fluconazole 400 mg OD for the next twelve months.

**Fig. 3:**
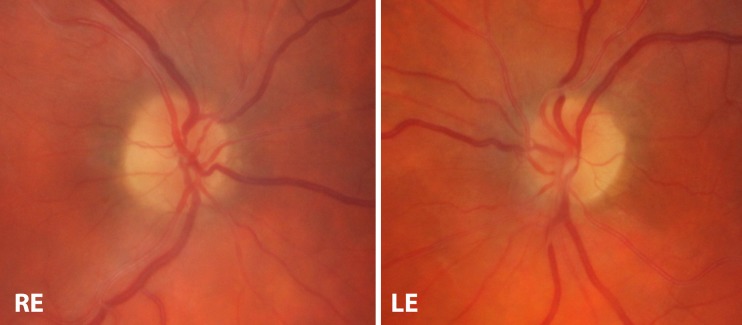
Colour fundus photograph showing resolution of left optic disc swelling and normal right fundus

The patient’s headaches improved completely after antifungal therapy, and visual acuity was measured at R 6/9 and L 6/9, Ishihara plate testing R15/17 and L 2/17. The left optic disc swelling also resolved (Fig. 3).

## Discussion

Cryptococcal infections (crypotococcoses) are most commonly caused by *C. neoformans*. This species is almost always synonymous with immunosuppression, particularly HIV infection. It has also been described with organ transplantation, malignancy, sarcoidosis, liver failure and even diabetes mellitus [2–4]. However, in a recent Canadian study, MacDougall et al. [5] reported the occurrence of infection in immunocompetent individuals by the *Cryptococcus gatii* species.

Asymptomatic pulmonary infection by *Cryptococcus* is common, even in the immunocompetent; usually, it is only in the immunocompromised that meningeal involvement occurs. Routine blood tests may be normal, making the diagnosis more difficult to confirm. Unfortunately, ‘cure’ can often prove difficult, with patients suffering from relapse of infection [6].

Due to our patient’s occupation as a farmer, he was frequently in contact with avian excreta, a potential source of infection by *Cryptococcus* species [7]. Furthermore, he was immunosuppressed, not only through receiving recurrent systemic corticosteroids but also through having sarcoidosis per se, which may have made him additionally vulnerable to the cryptococcal infection. There have been several reports of a specific association between cryptococcal infection and sarcoidosis [8–10]. Botha and Wessels [10] postulated that this susceptibility is either due to immunosuppression from steroid use or exclusively to impaired T cell-mediated immunity as a result of sarcoidosis in patients not treated with steroids. Impaired T cell-mediated immunity in such patients could be due to the sequestration of T cells within organs affected by sarcoid, such as the lung.

## Conclusion

The diagnostic challenge of differentiating an infectious cause from a non-infectious (inflammatory) cause is a common one when a patient presents for the first time with posterior uveitis. Non-infectious causes are much more common, and once confirmed, the patients usually respond well to immunosuppression with steroids or steroid-sparing agents. This case illustrates a rare and challenging scenario in which a patient presents with an infective cause of posterior uveitis but has had multiple episodes of non-infectious flare-ups of posterior uveitis which responded well to immunosuppression previously. Therefore, less common, infectious causes of active posterior uveitis need to be considered not only when investigating patients with their first presentation but also when they present with ‘flare-ups’ despite a previous history of non-infectious flare-ups as they can be immunocompromised from their therapy or from the underlying inflammatory conditions.
